# Potentially Common Therapeutic Targets for Multiple Sclerosis and Ischemic Stroke

**DOI:** 10.3389/fphys.2018.00855

**Published:** 2018-07-13

**Authors:** Roberto Paternò, Jean-Marc Chillon

**Affiliations:** ^1^Dipartimento di Medicina Clinica e Chirurgia, Università degli Studi di Napoli Federico II, Naples, Italy; ^2^Mécanismes Physiopathologiques et Conséquences des Calcifications Cardiovasculaires (EA 7517), Faculty of Pharmacy, University of Picardie Jules Verne, Amiens, France; ^3^Direction de la Recherche Clinique et de l’Innovation, CHU Amiens Picardie, Amiens, France

**Keywords:** multiple sclerosis, ischemic stroke, common pathway, common therapy, neuroinflammation

## Abstract

Ischemic stroke (IS) and multiple sclerosis (MS) are two pathologies of the central nervous system (CNS). At the first look, this appears to be the only similarity between the two diseases, as they seem quite different. Indeed IS has an acute onset compared to MS which develops chronically; IS is consecutive to blood clot migrating to cerebral blood vessels or decrease in cerebral blood flow following atherosclerosis or decreases in cardiac output, whereas MS is an immune disease associated with neurodegeneration. However, both pathologies share similar pathologic pathways and treatments used in MS have been the object of studies in IS. In this mini-review we will discuss similarities between IS and MS on astrocytes and neuroinflammation hallmarks emphasizing the potential for treatments.

## Introduction

Ischemic stroke (IS) and multiple sclerosis (MS) are two pathologies of the central nervous system (CNS). It has been proposed that the two pathologies may share similar pathological pathways involving particularly glutamate release, oxidative stress, and reactive oxygen species (ROS). During IS, activation of glutamate receptors following the release of this excitatory neurotransmitter leads to an increase in intracellular calcium and activation of nitric oxide synthase and NADPH oxidase pathways. The resulting increases in ROS contribute to neuronal death, increases in blood–brain barrier (BBB) permeability and ischemic lesion development (for review see Forrester et al., 2018). Similarly, glutamate may be involved in MS development. Oligodendrocytes, the myelin-producing cells of the CNS, are vulnerable to glutamate excitotoxicity via glutamate receptors activation. ROS generated following glutamate receptors activation may contribute to demyelination and neuronal degradation in MS (for review, see Gilgun-Sherki et al., 2004; Iodice et al., 2017).

## Astrocytes

### Astrocytes and Stroke

For a long time, glia cells were believed to be only structural cells. It is now well known that glia cells such as astrocytes or microglia play a role in the CNS functions in physiological and pathological conditions. Indeed, astrocytes regulate ion and neurotransmitters homeostasis, metabolically support neurons, and monitor synaptic activity (for review, see Parpura et al., 2012). Astrocytes glutamate transporters play a major role in glutamate clearance and excitotoxicity by removing glutamate from the extracellular space and maintaining it below neurotoxic levels (Rothstein et al., 1996). It has been reported that glial glutamate transporter (GLT-1) may have a dual effect in stroke by taking up glutamate and thus protecting neurons in the early stages of ischemia and by releasing glutamate and triggering neurons death with prolonged ischemia (Mitani and Tanaka, 2003). Furthermore, astrocytes form a functional syncytium thanks to gap junctions composed of the channel protein connexin43 (Giaume et al., 1991). Such syncytium may protect the CNS during IS by dispersing potassium or glutamate released from neuron in the extracellular space and accumulated by astrocytes. Indeed, infarct volumes were significantly increased in connexin43 heterozygous null mice compared to wild type mice (Siushansian et al., 2001). Reactive astrogliosis also occurs following CNS injury with beneficial and deleterious effects. Astrogliosis involves morphological and functional changes and contribute to glial scar that may protect preserved healthy tissues from inflammation but also may decrease ischemic tissue recovery by reducing axons regeneration. Actually, glial scar may be beneficial in the early stages of stroke allowing to limit lesion size but deleterious if not resolved by decreasing neuroplasticity and CNS regeneration (Pekny et al., 2014). Several factors such as p53 (Ahn et al., 2015), p38 mitogen-activated protein kinase (Roy Choudhury et al., 2014), macrophage-derived osteopontin (Gliem et al., 2015), acute-phase protein pentraxin-3 (Rodriguez-Grande et al., 2014), and CD36 (Bao et al., 2012) are involved in astrocytes activation (see Sofroniew, 2009 for more information).

### Astrocytes and MS

In MS, microglia activation has been shown in all clinical subtypes of the disease (Prineas et al., 2001), astrocytes produce a glial scar when inflammation decreases. In recent studies, the astrocytes are recognized as early and highly active players during MS lesion formation and as having beneficial and detrimental roles during MS lesion evolution. Studies indicate that astroglial myelin phagocytosis is an early event that takes place before the damaged myelin is removed by macrophages. Thus modulation of early astroglia responses can be a possible target for MS treatment (Ponath et al., 2017).

In MS, all aspects of glutamate homeostasis are impaired, indicating that glutamate excitotoxicity is an essential mechanism in the pathogenesis of the disease. Many studies have shown that glutamate levels are increased in the cerebrospinal fluid (CSF) (Sarchielli et al., 2003) and in acute lesions of MS patients (Srinivasan et al., 2005). There is an increase of glutaminase expression in macrophages and microglia in close proximity to dystrophic axons. In addition, in experimental autoimmune encephalomyelitis (EAE), an animal model of MS (Werner et al., 2001), there is a correlation between glutaminase expression and axonal damage.

Today, several mechanisms have been taken into account that may link astrocytic glutamate release with the glutamate excitotoxicity present in MS, and all these mechanisms involve microglia activation; in fact, after activation, these cells release adenosine triphosphate (ATP), which activates the P2Y1 receptor on astrocytes, leading to glutamate release (Pascual et al., 2012). Considering that in MS immunoinflammatory and neurodegenerative processes coexist, the glutamate excitotoxicity could be the missing link between them. This concept has practical value in developing innovative therapy that should take into account the immunosuppression as well as the neuroprotection.

### Astrocyte-Targeted Strategies

#### Astrocyte-Targeted Strategies in Stroke (**Table 1**)

**Table 1:** Common therapeutic targets for multiple sclerosis and ischemic stroke.

Targets	Molecular patterns	Consequences
Astrocytes	- Overexpression of heat shock protein 72	↑ astrocytes resistance to ischemic stress (Xu et al., 2010)
	- Overexpression of superoxide dismutase 2	↑ astrocytes resistance to ischemic stress (Xu et al., 2010)
	- Pyruvate	↑ glutathione (antioxidant) synthesis (Miao et al., 2011)
	- Ceftriaxone	↑ glial glutamate transporter (Ouyang et al., 2007; Verma et al., 2010)
	- Inhibition of p53	↓ astrocytes activation (Ahn et al., 2015)
	- microRNA-29a	↑ neurons survival during ischemia (Ouyang et al., 2013)
	- P2X7 receptors	↑ brain ischemic tolerance by preconditioning (Hirayama et al., 2015)
Blood–Brain Barrier	- Insulin growth factor 1	↑ BBB integrity (Bake et al., 2014)
	- Overexpression of heat Shock protein 27	↑ BBB integrity (Shi et al., 2017)
	- Preservation of tight junction by Sac-1004	↑ BBB integrity (Zhang H. et al., 2017)
	- Blockade of α4 integrin	↓ peripheral immune cells infiltration Becker et al., 2001; Investigators, 2001; Relton et al., 2001; Liesz et al., 2011b; Langhauser et al., 2014; Llovera et al., 2015; Elkins et al., 2017)
Neuroinflammation	- Fumarate	Immunomodulatory and antioxidant properties (Lin et al., 2016)
	- Fingolimod	↓ lymphocytes influx and thrombo-inflammation (Liesz et al., 2011a; Kraft et al., 2013; Fu et al., 2014)
	- Nrf2 activation	↓ microglia activation and CNS peripheral cells infiltration (Kuo et al., 2017)
	- IL1 inhibitor	↓ inflammatory cytokines expression (Zhang D.D. et al., 2017)
	- IL33	↑ anti-inflammatory Th2 responses (Luo et al., 2014; Korhonen et al., 2015)
	- IL4	Modulation of microglia activation (Korhonen et al., 2015; Xia et al., 2015; Zhao et al., 2015)
	- Protein kinases inhibitors	↑ M2-polarized microglia (Lee and Suk, 2017)
	- microRNA	↑ M2-polarized microglia (Ni et al., 2015; Hamzei Taj et al., 2016)
	- TNF-α	↓ inflammation (mixed results) (Sumbria et al., 2012; Clausen et al., 2014; Pires et al., 2014; Probert, 2015; Palle et al., 2017; Wu et al., 2017)
	- IL6	↓inflammation (Maimone et al., 1997; Beauchemin and Carruthers, 2016; Kleiter et al., 2016; Wang et al., 2016)

Astrocyte-targeted-strategies may be an option for stroke therapy. Increasing astrocyte survival during ischemic stress is associated with an increased neuronal survival. Indeed, astrocyte targeted overexpression of heat shock protein 72 and superoxide dismutase 2 increases astrocyte resistance to ischemic stress and preserves CA1 neurons following forebrain ischemia (Xu et al., 2010). Similarly, pyruvate increases the synthesis of glutathione, an antioxydant protecting cells from toxins such as free radicals. Pyruvate administration protects against glutamate-induced toxicity in mixed culture of cortex cells but not in pure neuronal cultures (Miao et al., 2011). Furthermore, addition of astroglia to the pure neuronal cultures restores pyruvate-associated neuronal protection (Miao et al., 2011). Other experiments indicate that upregulation of GLT-1 expression in astrocytes with ceftriaxone (Ouyang et al., 2007; Verma et al., 2010) or viral-mediated gene delivery (Weller et al., 2008) protects neurons from ischemia. Another potential target for stroke therapy is p53 as it has been shown that inhibition of p53 activity prevents astrocyte activation and astrocyte impaired glutamate intake (Ahn et al., 2015). Another potential targets for astrocytes and ischemic protection are microRNAs. MicroRNAs, some of them expressed in astrocytes, appear to be involved in the regulation of cerebral ischemia and may be targets to improve stroke outcome (Ouyang et al., 2014). Indeed increasing levels of microRNA-29a, a microRNA strongly expressed in astrocytes, protects neurons during forebrain ischemia (Ouyang et al., 2013). Finally, some experiments also suggest that astrocytes may be implicated in the induction of brain ischemic tolerance by preconditioning. This was linked to an upregulation of P2X7 receptors by astrocytes following preconditioning (Hirayama et al., 2015).

#### Astrocyte-Targeted Strategies in MS (**Table 1**)

The P2X7 receptor has also been implicated in the pathogenesis of MS. P2X7R immunoreactivity is increased in activated microglia/macrophages in spinal cord during MS (Yiangou et al., 2006). In addition, pharmacological inhibition of the receptor diminishes astrogliosis in rat EAE and reduces neurological symptoms (Grygorowicz et al., 2016). However, conflicting results have been obtained in this animal model of MS. In fact P2X7 receptor knockout mice are more resistant to EAE than wild-type mice (Sharp et al., 2008). On the other hand, in another study, the P2X7 receptor knockdown mice have a more severe EAE (Chen and Brosnan, 2006). Conflicting results were also observed regarding P2X7 receptors and IS. On one side, activation of P2X7 receptors appears necessary for inducing ischemic tolerance by preconditioning (Bindra et al., 2014; Hirayama et al., 2015) and attenuate brain edema after IS (Kaiser et al., 2016). On the other side, P2X7 receptors are involved in microglial cell (Eyo et al., 2013) and neuronal death (Arbeloa et al., 2012) during oxygen-glucose deprivation.

## Neuroinflammation

### Immune Cells Infiltration/Blood–Brain Barrier Dysfunction and Stroke

The brain initial innate response to stroke is essentially mediated by microglia, the resident macrophage of the CNS. This initial step is then followed by infiltration of immune cells such as neutrophils, macrophage/monocytes, T cells (Ma et al., 2017). In ischemic conditions, neurons release damage associated molecular patterns (DAMPs) leading to glial (microglia and astrocytes) activation and chemokines liberation. This will lead to endothelial cells activation, with expression of adhesion molecules allowing interaction between peripheral immune cells and endothelial cells followed by diapedesis (Strecker et al., 2017). Furthermore, microglia activation induces the production of ROS through the activation of NADPH oxidase associated with BBB disruption (Sumi et al., 2010). Other pro-inflammatory cytokines, such as tumor necrosis factor (TNF-α) and interleukin (IL)1β are also secreted by activated microglia and contribute to BBB dysfunction (Da Fonseca et al., 2014). Microglia can also express matrix metalloproteinase (MMP) following activation (Del Zoppo et al., 2007). MMP also play a role in BBB dysfunction during IS following degradation of tight junction proteins (Liu et al., 2012; Li et al., 2013). This may contribute to the deleterious role of MMP-9 in the development of brain injury after focal cerebral ischemia (Asahi et al., 2000). Finally, it has been reported that all microglia in the penumbra are associated to endothelial cells within 24 h post reperfusion and destroy endothelial cells by phagocytosis contributing to BBB disruption (Jolivel et al., 2015). It has also been reported that other cells than microglia, such as mast cells, are potentially initiators of BBB dysfunction and neuroinflammation (McKittrick et al., 2015). Peripheral immune cells infiltration following BBB disruption release anti-microbial enzymes, reactive oxygen/nitrogen species and chemokines responsible for further inflammation and BBB dysfunction (Strecker et al., 2017).

### M1 Phenotype Versus M2 Phenotype and Stroke

When activated, immune cells can acquire 2 phenotypes, M1 activated phenotype and M2 activated phenotype (Olah et al., 2011; Easton, 2013; Patel et al., 2013). The M1 phenotype is characterized by high expression of destructive pro-inflammatory mediators and contributes to ischemic lesions extension. In contrast, the M2 phenotype presents neuroprotective properties. Furthermore, M2 phenotype facilitates phagocytosis, thus reducing secondary inflammatory reaction and making space for newborn neurons. It has been observed that microglia and macrophages respond dynamically to ischemic injury with, first, an increase in the protective M2 phenotype followed by a transition to the pro-inflammatory M1 phenotype (Hu et al., 2012). Several factors may contribute to immune cells polarization. Ischemic neurons may contribute to M1 microglial activation by releasing soluble FAS ligand (Meng et al., 2016). In contrast, neurons in the penumbra produce IL4, a cytokine with the ability to polarize macrophages to the M2 phenotype (Zhao et al., 2015).

### M1 Phenotype Versus M2 Phenotype and MS

In both MS and experimental animal models of MS, intracerebral M1 phenotype cells (Broholm et al., 2004) as well as M2 phenotype (Boven et al., 2006) have been detected. Reactive microglia/macrophages exert both neurodestructive and neuroprotective effects in MS contributing to the most common clinical presentation of MS, the relapsing-remitting form. It has been observed that both M1 and M2 activation states can occur at the same time in EAE, and that the M1 to M2 ratio is a key factor in relapse of EAE (Miron et al., 2013). The M1 state is associated with progressive EAE whereas the M2 state may suppress the clinical symptoms of EAE (Ransohoff and Perry, 2009). It has also been observed that both MS and EAE are characterized by predominance of M1 microglia in the acute or early phase of the disease (Mikita et al., 2011). M1 markers appear in normal-appearing white matter and in active and inactive white matter lesions, whereas M2 markers are mainly expressed in the perivascular space (Zhang et al., 2011). For both stroke and MS, considering M1 and M2 polarization, the therapy must be developed to prevent excessive microglial activities but also to preserve their protective functions.

### Cytokines and Chemokines Involved in Stroke

In a recent review, Iadecola and Anrather listed mediators of post-ischemic inflammation. They separated mediators involved in the initiation, the amplification and the resolution of IS (Iadecola and Anrather, 2011). Considering cytokines, IL1α and IL1β and TNF-α are involved in the initiation of post-ischemic inflammation. Then, IL1, 6, 10, 17, 20, and TNF-α contributes to the amplification of the neuroinflammation whereas TGF-β, IL10, 17, and 23 contribute to its resolution (Iadecola and Anrather, 2011). Numerous chemokines such as CCL5, CXCL4, CXCL7, CX3CL1, (initiation) and CCL2, CCL3, CCL5, CXCL2/3, and CXCL8 (amplification) also contributes to post-stroke inflammation (Iadecola and Anrather, 2011). Other mediators include adhesion molecules, proteases, and small molecules such as prostanoids and leukotriens for initiation and iNOS, C0X-2 and NADPH oxidase for amplification (Iadecola and Anrather, 2011).

### Neuroinflammation-Targeted Strategies

#### Neuroinflammation-Targeted Strategies and Stroke (**Table 1**)

Considering the importance of neuroinflammation in ischemic stroke, immunomodulation appears like an interesting therapeutic option. Immunomodulation is currently used for MS treatment and several drugs used in MS have been evaluated in ischemic stroke. Fumarate, because of its immunomodulatory and antioxidant properties, suppresses pro-inflammatory cytokines in *in vitro* and *in vivo* stroke models. This is associated with a decrease in infarct size and an improvement in behavioral outcome (Lin et al., 2016). In animal models of ischemic stroke, treatment with fingolimod is associated with mixed results. In one study, fingolimod reduced post-stroke lymphocytes influx but had no favorable impact on infarct volume and behavioral dysfunction (Liesz et al., 2011a). In another study it has been shown that fingolimod has stroke-protective action by reduction of thrombo-inflammation but not by a direct neuroprotective effect (Kraft et al., 2013). Furthermore, in a small clinical trial, oral fingolimod within 72h of stroke onset was associated with decreased microvascular permeability, attenuated neurological deficits and improved recovery (Fu et al., 2014). Few papers on the impact of glatiramer in IS are available. The results are somewhat contradictory with either no reduction of infarct volume or improvement in neurological deficit in mice (Poittevin et al., 2013; Kraft et al., 2014) or an improvement of neurological deficit and an increase in neurogenesis and decrease in infarct volume in rats (Ibarra et al., 2007; Cruz et al., 2015).

Other treatments modulating directly or indirectly neuroinflammation may present an interest for stroke therapy. Indeed, it has been reported that activation of Nrf2 is associated with a decrease in microglia activation and CNS peripheral cell infiltration as well as a protection against IS in mice (Kuo et al., 2017). It has also been shown that a fusion protein, which fused the natural inhibitor of IL1, the IL1 receptor antagonist, with a cell penetrating peptide, alleviates brain infarction, cerebral edema, neurological deficit score, motor performance and inflammatory cytokines expression (Zhang D.D. et al., 2017). Several IL may also present an interest. For example, IL33 is protective against ischemic insult by promoting the anti-inflammatory Th2 responses (Luo et al., 2014; Korhonen et al., 2015). This protective effect seems also related to the induction of IL4 secretion (Korhonen et al., 2015). It has been previously reported that IL4 is secreted by ischemic neurons as an endogenous defense mechanism by modulating microglia activation (Zhao et al., 2015). Indeed, M2-polarized microglia with its anti-inflammatory profile is a promising therapeutic option for stroke therapy (Xia et al., 2015). Other potential targets to decrease microglia-mediated neuroinflammation by increasing M2-polarized microglia are protein kinases inhibitors (Lee and Suk, 2017) or microRNA such as let-7c-5p (Ni et al., 2015) or microRNA-124 (Hamzei Taj et al., 2016). As, as previously mentioned, neuroinflammation involves infiltration by peripheral immune cells of the CNS, other potential therapies for stroke prevention may be to decrease such infiltration. Decreasing BBB disruption may do this. Indeed, preservation of the BBB integrity by insulin growth factor 1 (Bake et al., 2014), overexpression of heat shock protein 27 (Shi et al., 2017), or preservation of tight junction by Sac-1004 (Zhang H. et al., 2017) is associated with an improved post-stroke neurological outcome. Another possibility to decrease peripheral immune cells infiltration is to modulate adhesion molecule. Blockade of α4 integrin can protect the brain against ischemic injuries in experimental models of IS (Becker et al., 2001; Relton et al., 2001; Liesz et al., 2011b). In contrast, blockade of α4 integrin was ineffective to protect from acute IS (Langhauser et al., 2014). This apparent discrepancy may be explained by the results of a preclinical multicenter trial on anti CD49d treatment for acute brain ischemia (Llovera et al., 2015). Indeed, treatment with CD49d-specific antibodies reduced leukocytes invasion and infarct volume in stroke model associated with small cortical infarction but not in stroke model associated with large ischemic lesion suggesting that treatment efficacy may depend on infarct severity or localization (Llovera et al., 2015). In humans, anti ICAM-1 therapy worsened stroke outcome in a clinical trial (Investigators, 2001) whereas treatment with natalizumab, an α4 integrin blocker, did not reduce infarct growth but had a beneficial effect on functional outcome (Elkins et al., 2017). Noteworthy, natalizumab is currently used as MS treatments and its use in experimental models of IS once again emphasizes the similarities between the 2 pathologies.

Few data are available on the impact of TNF-α blocking on IS lesions. It has been observed that patients with psoriasis and treated with TNF-α inhibitors had a lower cardiovascular event risk compared to patients treated with phototherapy (Wu et al., 2018) or methotrexate (Wu et al., 2017). Furthermore, anti TNF-α therapy ameliorates functional outcomes after stroke by altering the peripheral immune response but without any impact on infarct volume (Clausen et al., 2014). One of the potential problems for IS treatments with anti TNF-α therapy may be BBB crossing. Indeed engineering of a BBB crossing TNF-α inhibitor allowed to decrease infarct volume and to improve neurological outcome in a stroke experimental model (Sumbria et al., 2012). However, we have to remain cautious and more data are necessary on the impact of TNF-α inhibitors on IS as it has been observed that TNF-α inhibition was associated with increased ischemic damage following a decrease in innate immune response from the brain (Pires et al., 2014). Considering IL6, it has been reported that pre-treatment with tocilizumab, a monoclonal antibody against IL6 in a rat model of ischemic stroke, prevents neuronal cell apoptosis (Wang et al., 2016).

In conclusion, targeting of neuroinflammation appears a promising option in IS treatment. Several clinical trials have been performed or are underway to evaluate clinical outcome in patients (for review see (Veltkamp and Gill, 2016)).

#### Neuroinflammation-Targeted Strategies and MS (**Table 1**)

It is well known that MS is an immume disease and the scope of this review is not to describe inflammatory pathways involved in MS. Most if not all of MS treatments are immunomodulatory or immunosuppressive drugs. In this field, several inflammatory factors may be identified as new potential targets for MS treatment. Indeed, several studies show that cytokines contribute to the pathogenesis of MS. Indeed, increased levels of TNF-α can be found in active lesions within the CNS as well as in the serum and CSF of MS patients (Wen et al., 2012). Furthermore, increased levels of TNF-α in CSF are in relation with the severity and progression of the disease (Sharief and Hentges, 1991). Thus studies have been done in mouse models of MS to test treatment strategy aiming to block TNF-α with very encouraging results (Palle et al., 2017). The hypothesis that neutralization of TNF-α may reduce or arrest MS progression was evaluated in a phase II randomized, multicenter, placebo-controlled study using lenercept, a recombinant TNFR1 fusion protein. However results were discouraging as patients treated with lenercept suffered from increased disease activity. These results suggest that non-selective blockade of TNF-α is detrimental (The Lenercept Multiple Sclerosis Study Group and The University of British Columbia Ms/Mri Analysis Group, 1999). One possible explanation could be that TNF-α may exert both proinflammatory effects and protective functions, for example, mediating remyelination in the CNS under pathological conditions (Probert, 2015). Other cytokines could be involved in the pathogenesis of MS as increased levels of IL6 have been found in active plaques of individuals suffering from MS (Maimone et al., 1997). It has also been reported that IL6-deficient animals were fully resistant to EAE (Kleiter et al., 2016). However, we have to remain cautious as a report described a patient with rheumatoid arthritis who developed MS during anti-IL6 therapy (Beauchemin and Carruthers, 2016). Furthermore, it has been reported that patients treated with anti-TNF-α therapies are at risk to develop clinical signs of MS. Putting all these data together we can speculate that IL6 may have beneficial effect in patients with MS.

## Conclusion and Perspectives

**FIGURE 1:**
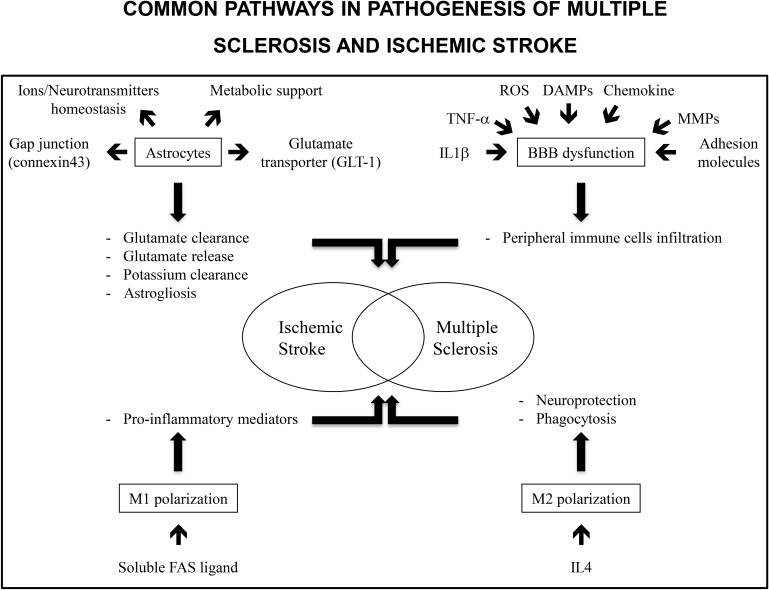
Common pathways in pathogenesis of multiple sclerosis and ischemic stroke include astrocytes, BBB dysfunction and M1/M2 polarization. Astrocytes are involved in ions and neurotransmitters homeostasis and metabolic support. Gap junctions as well as glutamate transporters allow for glutamate uptake and clearance. Similarly, gap junctions allow for potassium clearance. Stored glutamate may be released by astrocytes and may be responsible for delayed lesions. In the same way astrogliosis has deleterious and protective effects. It protects preserved tissue from inflammation but decreased neurogenesis in ischemic tissue. Inflammatory markers such as TNF-α or IL1-β, reactive oxygen species, danger associated molecular patterns (DAMPs, molecules released from necrotic cells), chemokines, matrix metalloproteinases (MMPs), and adhesion molecules expression contribute to BBB disruption and peripheral immune cells infiltration. M1 polarization of immune cells is associated with an increase in pro-inflammatory mediators and deleterious effects on neuronal lesions. In contrast, M2 polarization is associated with neuroprotection and dead cells phagocytosis allowing for a decrease in inflammation.

IS and MS share common pathological pathways such as astrocytes activation, BBB disruption and microglia/macrophages polarization (**Figure 1**). This allows emphasizing similar treatments. Already, drugs commonly delivered for MS treatment have been the object of clinical trials for IS care using their immunomodulation potential. Potential innovative treatments targeting astrocytes activation, BBB integrity and neuroinflammation with modulation of microglia/macrophages polarization or cytokines expression have been the subjects of animal studies and clinical trials (**Table 1**). This is of interest as, except for thrombolysis, no treatment has been identified to decrease IS burden.

## Author Contributions

RP conceived and wrote the article. J-MC wrote the article.

## Conflict of Interest Statement

The authors declare that the research was conducted in the absence of any commercial or financial relationships that could be construed as a potential conflict of interest.
